# Suppressed or unsuppressed HIV in adults on antiretroviral therapy in Zambia: who is at risk?

**DOI:** 10.7448/IAS.15.6.18100

**Published:** 2012-11-11

**Authors:** N Chishinga, P Godfrey-Faussett, K Fielding, A Grant, A Schaap, H Ayles

**Affiliations:** 1 ZAMBART Project, Lusaka, Zambia; 2 London School of Hygiene and Tropical Medicine, Clinical Research, London, UK; 3 London School of Hygiene and Tropical Medicine, Infectious Disease Epidemiology, London, UK

## Abstract

**Purpose of the study:**

To determine factors associated with suppressed or unsuppressed HIV in adults receiving combination antiretroviral therapy (cART) in Zambia.

**Methods:**

This was a cross-sectional study conducted between August 2008 and October 2009 in 16 Zambian communities nested within the ZAMSTAR trial [1]. Adult TB cases identified at a TB clinic of each community and their adult household members were invited to participate in the study. A structured interview was used to obtain information on the participants’ social, demographic and clinical characteristics. Socio-economic position (SEP) was measured using household wealth indices used in demographic health surveys. Principal component analysis was used to determine the cut-off for high (wealthy) and low (poor) SEP. Depression symptoms were measured using the Center for Epidemiological Studies Depression scale (CES-D). A cut-off of≥22 on the CES-D was used to define current depression [2]. Participants were included in this analysis if they were found to be receiving cART for>90 days at the time of the interview. The outcome was HIV suppression (viral load≤300 copies/ml). In both univariable and multivariable analyses, log Poisson regression models with robust standard errors adjusted for the 16 communities were used to calculate the risk ratios (RR), 95% confidence intervals (CI) and p-values of factors associated with HIV suppression. In multivariable analysis, each variable was independently assessed for its association with HIV suppression while minimally adjusting for *a priori* confounders (age, gender and education level).

**Summary of results:**

There were 520 patients receiving cART for>90 days. The median age was 35 years (inter-quartile range: 31–41) and 328/520 (63.1%) were married (Table).

Of the 520 patients, 442 (85.0%) had HIV suppression while 78 (15.0%) did not. At univariable analysis, having high SEP was negatively associated with HIV suppression while receiving ZDV+3TC+EFV was positively associated with HIV suppression. At multivariable analysis, patients with high SEP were less likely to have HIV suppression than those with low SEP.

**Conclusions:**

Patients with high SEP were found to be at risk of having unsuppressed HIV. There is need for targeted interventions that can improve HIV outcomes in this group of patients receiving cART in Zambia.

**Table:** 

		HIV suppression		HIV suppression vs. no suppression			
			
	n=520	Yes	No	Unadjusted		Adjusted	
					
	N (column %)	N (row %)	N (row %)	RR (95% CI)	p-value	RR (95% CI)	p-value
**Age group, years**							
16 to 25	47 (9.0)	39 (83.0)	8 (17.0)	1			
26 to 35	220 (42.3)	183 (83.2)	37 (16.8)	1.00 (0.85–1.18)	0.976		
36 to 45	187 (36.0)	162 (86.6)	25 (13.4)	1.04 (0.91–1.20)	0.541		
Above 45	66 (12.7)	58 (87.9)	8 (12.1)	1.06 (0.90–1.25)	0.503		
**Gender**							
Men	204 (39.2)	169 (82.8)	35 (17.2)	1			
Women	316 (60.8)	273 (86.4)	43 (13.6)	1.04 (0.97–1.12)	0.261		
**Education level**							
None/Primary	241 (46.3)	200 (83.0)	41 (17.0)	1			
Secondary	279 (53.7)	242 (86.7)	37 (13.3)	1.05 (0.95–1.15)	0.358		
**Socio-economic position (SEP)**							
Low	287 (55.2)	252 (87.8)	35 (12.2)	1		1	
High	220 (42.3)	177 (80.5)	43 (19.5)	0.92 (0.86–0.98)	0.009	0.90 (0.84 – 0.96)	0.001
Missing	13 (2.5)	12 (92.3)	1 (7.7)				
**Type of cART**							
Tenofovir (TDF)+emtricitabine (FTC)+nevirapine (NVP)	61 (11.7)	48 (78.7)	13 (21.3)	1		1	
TDF+FTC+efavirenz (EFV)	182 (35.0)	154 (84.6)	28 (15.4)	1.08 (0.95–1.22)	0.26	1.09 (0.96–1.25)	0.187
Stavudine (d4T)+lamivudine (3TC)+NVP	197 (37.9)	168 (85.3)	29 (14.7)	1.08 (0.97–1.21)	0.158	1.05 (0.94–1.18)	0.379
Zidovudine (ZDV)+3TC+EFV	78 (15.0)	70 (89.7)	8 (10.3)	1.14 (1.01–1.28)	0.027	1.11 (0.99–1.25)	0.071
Missing	2 (0.4)	2 (100.0)	0 (0.0)				
**History of tuberculosis**							
No	108 (20.8)	92 (85.2)	16 (14.8)	1		1	
Yes	412 (79.2)	350 (85.0)	62 (15.0)	1.00 (0.89–1.11)	0.961	1.01 (0.91–1.13)	0.818
**Current depression**							
No	416 (80.0)	355 (85.3)	61 (14.7)	1		1	
Yes	77 (14.8)	63 (81.8)	14 (18.2)	0.96 (0.84–1.09)	0.523	0.96 (0.84–1.09)	0.493
Missing	27 (5.2)	24 (88.9)	3 (11.1)				
